# Lectures on Diseases of the Skin

**Published:** 1855-12

**Authors:** Austin Flint

**Affiliations:** Professor of the Theory and Practice of Medicine in the University of Louisville, Ky.


ART. II.—*Lectures on Diseases of the Skin.* By AUSTIN FLINT, M. D., Professor of the Theory and Practice of Medicine in the University of Louisville, Ky.

LECTURE II.

GENTLEMEN: In the diagnosis of cutaneous affections, the first question which arises whenever a case comes under notice, is, to which of the eight orders does it properly belong. Having the distinctive character of each order before the mind, we are to determine whether, on the one hand, those distinctive of any one of the orders are present, and, on the other hand, those distinctive of the other orders are absent. Here, as in the diagnosis of other affections, we are to take into view positive and negative facts; in other words, to reason from what is actually observed, and, by way of exclusion, also from what we do not observe. One source of difficulty in the diagnosis lies in the fact that the disease generally has existed for a greater or less period, and it is not always easy to determine what were the characters presented at its development, or early stage. In the progress of cutaneous affections not only do they come to resemble each other in their external characters, but, in some instances, they actually verge one into another, or become complicated by union with each other. Thus vesicles not only become in appearance, at least, pustules, but, after a time, pustules may be developed as such from the beginning, so that the affection is in reality both vesicular and pustular. It is important in such cases to obtain from the patient, or intelligent friends, as good a description as practicable, of the earliest appearances of the eruption. Without giving leading questions, we are to endeavor to ascertain the form which the affection at first manifested, whether water pimples, pus pimples, solid pimples, etc. It is usually the case that fresh developments of the original form of the eruption successively occur from time to time, so that by careful examination, and perhaps waiting a few days, the primitive or elementary characters are observable. The late Dr. Worcester, of Cincinnati, who compiled an excellent treatise on diseases of the skin, directed attention to a practical point which will often assist in locating, in their proper orders, cases in which, from long continuance, the distinctive characters of the affection are obscured. The point to which I refer, relates to the disease having been in its early stage *moist* or *dry*. This can generally be ascertained with certainty, and without much difficulty, when it might be unsafe to rely on the descriptions by the patient or friends of the particular appearances which the eruption presented. The *squamæ*, *papulæ*, and *maculæ* are *dry*, while the *vesiculæ*, *bullæ*, and *pustulæ* are *moist* eruptions. In other word, a liquid exudation characterizes the latter, and is absent in the former. Much importance is attached to this point by M. Devergie, one of the physicians at St. Louis Hospital, Paris. He makes it, indeed, the basis of a grand division of cutaneous affections into two classes, viz: *secreting* and *non-secreting*, the first embracing moist, and the latter dry eruptions. In a conversation with the highly intelligent *interne* in M. Devergie's wards, M. Fremineau, he claimed this division as an original feature of the work by M. Devergie, recently published. The treatise, by Dr. Worcester, was published ten years ago. Whether the point was original with Dr. W. I am not prepared to say, but, at all events, I had availed myself of it in practice, and been in the habit of inculcating its value in clinical instructions (being indebted for the suggestion to Dr. Worcester's treatise) for many years before I enjoyed the opportunity of studying skin diseases at St. Louis Hospital in the summer of 1854, the date of the publication of M. Devergie's work.

The discrimination of cutanéous eruptions does not rest solely on the primitive characters which are peculiar to each. Certain distinctive traits also pertain to the secondary appearances which they respectively present. Knowledge of these is therefore highly important with reference to diagnosis.

Some forms of cutaneous disease evince predilection for certain ages, and localities of the body. Some, in addition to appearances addressed to the eye, or characters ascertained by the touch, have a peculiar odor. The latter is said to characterize *favus* for example. Some are communicable by contact, in other words contagious, while the majority are never extended or reproduced in that mode. The presence of a peculiar insect distinguishes one species; and several species, it has been lately ascertained, are distinguished by the production of a cryptogamous plant proper to each. All these circumstances enter into the diagnostic criteria of the different forms of cutaneous disease.

It is seldom very difficult to trace an affection to the order to which it belongs. After this, the diagnosis relates to the localization in its proper genus, species and variety. To determine the genus, ordinarily does not occasion much trouble, with the advantage of a tolerable acquaintance with the subject; and to determine the species and variety is frequently of little practical consequence. It is in the diversity of species and varieties instituted by dermatological writers, that the charge of needless refinement and complexity is mainly admissible—but this topic we will defer till we can speak of it in connection with the respective orders of their subdivisions; and we will now proceed to treat severally of these.

Taking up the several classes in the order in which they have been enumerated (Sec. 1st,) the first which presents itself is the class of vesicular eruptions, or the *vesiculæ.*

The generic subdivisions of the *vesiculæ* are four in number, named as follows: *Eczema, Herpes, Scabies, Miliara.* Rayer enumerates an additional genus consisting of the vesicular eruptions occasionally produced by mercurials. He calls them *Hydrargyria*, and divides them into three species, which he designates *Hydrargyria mitis, febrilis*, and *maligna.* He has only met with three cases. It is quite unnecessary to burthen the memory with these names and distinctions. It is only desirable to bear in mind that a vesicular affection has been known to be occasioned, apparently, by mercury. If Rayer, with his large experience, has met with but three cases, it must be rare at least in France. It may possibly occur oftener in countries in which mercurial remedies are employed more indiscriminately, and with greater freedom.

*Eczema*, the name of the first of the four genera comprising the different forms of vesicular eruptions, has a Greek derivation signifying heat or effervescence. A popular epithet applied to the affection which it designates is *tetter.* The French use a term synonymous with tetter, viz: *dartre.* Eczema, however, is only one of various eruptions included within the popular appellation of the *tetters.* Some of its varieties have received, in some parts of our country, the title of *epidemic itch.* Other varieties are familiar to medical men by the name of *crusta lactea*, or, vulgarly, *milk crust.*

Eczema is characterized by minuteness of the vesicles. They are always very small, and closely crowded together, forming irregularly defined patches of greater or less size, sometimes with, and sometimes without a reddened or inflamed base. The minuteness of the vesicles is sometimes so great that it is difficult to distinguish them with the naked eye, and the common pocket microscope is useful in demonstrating their presence. In form they are orbicular and flattened, not acuminated or sharp pointed.

The contents of the vesicles, at first transparent, shortly becomes opaque. They may be absorbed, in which case the eruption disappears without much moisture, the walls of the vesicles being thrown off, followed by more or less subsequent desquamation of the cuticle. But generally a rupture of the vesicles takes place, and their contents escape, and by concretion and desiccation crusts are formed, giving rise to the secondary appearances of the eruption. Coalescence of the vesicles frequently takes place; the eruption becomes confluent, leading to excoriation of the surface, which may furnish a constant exudation of sero-puruloid matter, concreting into laminated scabs. In some instances superficial ulceration succeeds. Successive eruptions generally occur, so that after it has continued for some time, all the stages of the affection may be presented in the same case at the same moment. The disease may be of brief duration, or persist for an indefinite period. It may be simple or diversified, complicated or uncomplicated.

With this general description I will proceed to call your attention, first, to several circumstances pertaining to the natural history of the disease which are distinctive; and next, to those on which are based subdivisions into species and varieties.

In the majority of instances, before cases of eczema come under our observation, rupture of the vesicles has taken place, and we find the part or parts affected more or less covered with incrustations. These incrustations, except in one variety of the disease, and provided it be uncomplicated and the eruption have not given place to an ulcerated surface, are of a dirty white, or ashy color, not yellow, and not thick and laminated like *scabs* formed by the concretion of purulent matter, from which they are to be distinguished. On the other hand, they differ from the mass of epidermic scabs occurring in squamous affections, to which they bear sometimes a close resemblance. They adhere to the surface with considerable tenacity, except at their margins which are generally detached. This circumstance distinguishes them from the scabby crusts succeeding pustules (in impetigo); and the scaly crusts of a squamous eruption (psoriasis). In both the affections just mentioned, the crusts are more loosely adherent, being easily detached. The base of the eczematous patches is usually reddened, and the redness extends for a greater or less distance around the patch. The redness in the latter situation is insensibly lost in the surrounding whiteness of the skin. It is gradually shaded off, not ending abruptly, having a well defined border like other eruptions in which an areola surrounds the affected parts, *e. g.*, herpes, psoriasis. The redness at the base of the patches persists after the parts have healed. The surface then presents a smooth and polished aspect. In this respect, it differs from the pustular affection impetigo, to which, in some characters, it bears a resemblance. In consequence of the excoriation or slight ulceration which frequently ensues, a superficial cicatrix is left after the parts are healed. In this particular it differs from ordinary impetigo.

The liquid contents of the vesicles are at first transparent like water, or citron colored. It differs from water, or the serum of the blood, however, in being more viscous, admitting of being drawn out into threads. Linen saturated with it is stiffened as when soaked in gum water or starch. These circumstances distinguish it from pus. The liquid is alkaline, and possesses irritating properties.

If the excoriated surface be closely examined after the incrustations are removed, it is found to be studded with numerous red points. These are seen distinctly, on close inspection, with the naked eye, but more satisfactorily with a pocket magnifying glass. Frequently small drops of liquid may be observed to ooze from these minute *puncta*. Each of these points is, in fact, the base of a vesicle which has been ruptured, and its walls exfoliated; and it is from orifices opening here that the exudation escapes which furnishes the matter of the solid incrustations. The appearances just described are distinctive of eczema, and, in fact, are sufficient of themselves for the diagnosis.

Eczematous patches are very slightly tumefied. This is a circumstance somewhat distinctive. An exception to this rule obtains when the affection is seated on the ear.

The pruritus is usually a distressing symptom. It is tingling, burning, and frequently intense, and constant whenever the attention is not strongly diverted. The desire to rub, and scratch the affected parts i- irresistible, but adds to the local irritation without effecting even temporary alleviation.

If the affection persist in the same situation for a long time, fissures or cracks are apt to occur, attended with more or less escape of blood. These circumstances serve to modify the secondary appearances.*

The subdivision of eczema into species and varieties is based on deviations from the ordinary characters in acuteness, duration, situation, extent of surface affected, and combination with the characters of other cutaneous affections. The point of departure for the subdivision, is the mildest and simplest form of the disease, called *eczema simplex*. In eczema simplex the eruption is in the skin at the base of the agglomerated vesicles. The contents of the vesicles, if not absorbed, concrete into a thin incrustation, very little or no excoriation succeeding, and the affected surface is healed in a few days. In this, its most simple form, however, the eruption is apt to be reproduced, successively, either in the same part, or in different portions of the body, and, in this way, the disease may be perpetuated for a greater or less period.

---

* The varied appearances presented in this, as well as other eruptive diseases, were illustrated in the lecture-room, by plates, and the excellent models of THIBERT.

*Eczema rubrum* is the designation applied to a species characterized by notable redness of the surface on which the eruption appears, the redness preceding the development of the vesicles. In this species the distinctive feature consists in the addition, as a prominent element of the local affection, of erythema of the skin; and this element, it will be observed, precedes the appearance of the vesicles. This, like simple eczema, may run a short career, but is more apt to persist, and eventuate in the chronic form of the disease.

*Acute eczema* is another species, differing from that last mentioned in the fact that the part or parts in which the eruption is seated present, in addition, the characters of true inflammation. In erythema rubrum the affected parts present an inflammatory aspect, but the redness does not necessarily involve more than hyperæmia. It may be unattended by acuteness of symptoms, either local or general. In acute eczema, on the other hand, the surface not only presents an inflamed aspect, but the morbid actions are more intense. It is also frequently associated with some febrile movement, and general disorder of the system, which is not true, as a general remark, of other species of the disease. Moreover, in eczema rubrum the erythema exists antecedently to the vesicles, while in acute eczema the inflammatory condition is developed simultaneously with the vesicles. These circumstances suffice to mark the distinction between the two as distinct species of the disease.*

The vesicles, in some instances, rapidly assume the characters of pustules, in other words, their contents quickly become opaque or puruloid, and under these circumstances it is not always easy at once to determine whether the eruption be primitively or essentially vesicular or pustular. The fact shows that a relationship exists between these two classes of eruptions: and this relationship is expressed in the name by which the species thus characterized is known, viz: *eczema impetiginodes.* The latter term is derived from the name of a genus of the order pustulæ, viz: *impetigo.* Eczema impetiginodes is not, strictly speaking, a combination of the two affections eczema and impetigo. The eruption is at first vesicular, but the contents of the vesicles very quickly assume the appearance of a purulent fluid. It does happen, however, not infrequently in the progress of eczema, that true primitive pustules become intermingled with the vesicles. This is apt to be the result of

---

* Eczema rubrum is, in fact, a combination of eczema and erythema, the latter taking precedence in point of time.

mechanically irritating the affected parts by scratching, or otherwise; and of the injudicious use of certain local applications.

These varied forms of eczema are frequently more or less associated in the same case, at the same time. Even the different portions of a single eczematous patch sometimes present illustrations of the different species of this affection: in one portion representing eczema simplex; in another eczema rubrum; in another acute eczema, and in another eczema impetiginôdes.

In a large proportion of the cases which come under the observation of the medical practitioner, eczema has either become, or it eventuates in a chronic affection. Fresh vesicles appear from time to time; the excoriated surface left by the coalescence and rupture of the vesicles, furnishes an exudation, which, from its irritating properties, tends to keep up, and enlarge the boundaries of the local disease. The skin takes on an ulcerated condition; or it presents a raw, softened aspect retaining the impression of linen cloths if applied to it. Fissures or cracks take place, giving rise to more or less hæmorrhage. If the parts become partially healed, the new surface is smooth, red, and glistening, and a fresh eruption speedily breaks out. Thus it continues for weeks, months, years, and sometimes during the remainder of life. Of the many cases which I saw at the St. Louis Hospital, in most the affection had existed for several months, and in one instance for the space of ten years.*

Varieties of this affection are based on differences of locality. It receives from dermatologists different names according to the part of the body on which it is seated. There is scarcely any portion of the cutaneous surface which is exempt from a liability to this eruption, but some portions are more predisposed to it than others, and its tendency to particular situations varies in different periods of life. It frequently occurs on the face and scalp, constituting *eczema faciei* and *eczema capitis.* In infancy and childhood, and in females, it evinces a preference for these localities. It appears very often behind the ears, and in this situation swelling of the parts affected is more marked than in any other situation. Situated on the head and face it is commonly known as *crusta lactea,* or milk crust. It is to be distinguished, when seated on the scalp, from *favus*, an affection embraced in the order *pustulæ,* from which it essentially differs. The hair does not fall in eczema as in favus, and it is not, like the latter affection, highly contagious. For these

---

* M. Devergie gives the following statistics relative to the duration : Of 561 cases, the disease lasted 1 month in 19 ; 2 months in 28 ; from 2 to 6 months in 106 ; from 6 months to 1 year in 51 ; more than 1 year in 292 ; more than 10 years in 57.

reasons, and also with reference to appropriate measures of treatment which are quite different in the two diseases, it is highly important to make the discrimination in practice. This may always be done, although in some of the external, superficial characters, there is a resemblance between the two. It will be more convenient, however, to point out the means of discrimination in treating of favus. In adults, and especially in aged persons, it is apt to attack the legs; and it is frequently associated with varicose veins.

The scrotum is sometimes the part affected. Situated here it is apt to be particularly rebellious to medical treatment. The exudation is copious; the parts are irritated by the friction incident to walking, and very great annoyance is occasioned by the burning heat and pruritus. Occurring on the hands, between the fingers and upon the wrists, it is liable, in its simple form, without due knowledge or care, to be confounded with scabies or itch, an unfortunate mistake in several points of view. Acquaintance with the distinctive characters of each eruption, will, with proper attention, enable the practitioner to avoid the error of confounding them. Frequently the affections appear to be determined to the hands by the action of external irritating causes. Thus it is observed in bakers whose hands are constantly covered with flour, and in grocers who handle sugars, &c. Hence the names that have been applied to eczema in this situation, viz: grocer's itch and baker's itch. Washerwomen are liable to it from having their hands constantly wet. The following fact shows the connection between the local affection and the action of an external local cause; if the affection occur in a person whose hands are in contact with an irritating powdered substance, the eruption will generally be most abundant between the fingers, because the powdered substance accumulates and remains longer in contact with the skin in that situation; but if the irritating substance be a liquid, the dorsal surface of the hands will be most likely to be affected. This fact was fully illustrated by the cases under observation at the St. Louis Hospital. Chronic eczema situated on the hands, as well as other forms of cutaneous eruption, generally goes by the name of *salt rheum.*

Other parts of the body for which the affection has a preference are the vicinity of the umbilicus shortly after birth; the neighborhood of the nipple in the female; also the vulva, and in both sexes around the anus. In the two latter situations, viz: the anus and vulva, it is apt to be peculiarly obstinate and distressing.*

---

* M. Devergie *(traité pratique des maladies de la peau)* gives the following statistics respecting the locality of this affection: Of 600 cases, it was seated on the legs

Eczema is sometimes complicated with other cutaneous affections. I have seen it associated with the papular eruption called lichen; with the squamous disease, psoriasis, and with rupia, one of the genera of the order *bullæ.* In each of these instances the affection presents mixed characters, those which are primitive and predominant belonging to eczema, and the others to the affection existing as a complication. I can only make this general statement here. To give the characters of the different eruptions existing in combination with those of eczema would involve an anticipation of subjects to be treated of hereafter.

Finally, eczema is generally local; that is, a portion, or disconnected portions of the cutaneous surface only are affected. It usually, as already stated, extends over patches of the skin, of greater or less size. The patches affected are rarely regular in form. They may be large or small. A single patch only may exist, or it may be seated in different parts of the body at the same time. The disease, however, may be general; in other words, it may extend over the entire surface of the body, excepting the soles of the feet and the palms of the hands. Instances of this variety are extremely infrequent.

I have thus mentioned the more prominent of the circumstances entering into the natural history of eczema; and, of the several species and varieties of the disease, I have noticed those which it is of practical importance for the physician to recognize and recollect. Let us now rehearse, in a few words, the distinctive traits which are diagnostic. It is a moist eruption. If examined early it is found to present minute vesicles, scarcely visible to the naked eye, closely crowded together; and at different stages new vesicles are frequently appearing which are typical of the order and genus, and by which it is to be discriminated from other eruptions. But these criteria are not always available. The diagnosis must then be based on the secondary appearances. These offer certain points which are highly distinctive, viz: an exudation, gummy, capable of being drawn into threads, stiffening linen, thin, ashy-colored crusts adhering with considerable firmness except at their margins; numerous red points dotting an excoriated surface, and frequently drops of liquid standing upon them which have just exuded; an areolar redness insensibly lost in the white color of the skin; slight swelling; a burning pru-

---

in 446; on the forearm in 155; on the arm in 133; on the face in 114; on the scalp in 86; on the genitals in 68; on the neck in 63; on the chest in 54; on the abdomen in 50; and on the back in 45. These statistics were collected in the hospital St. Louis, where children are not received. This fact will explain the relatively small number of cases in which it was seated on the face and scalp.

ritus; persisting redness, and sometimes a superficial cicatrix after healing is completed. These few diagnostic criteria, taken in connection with negative points, that is, the absence of characters distinctive of other eruptions with which this is liable to be confounded, will suffice for a positive and easy diagnosis in a large proportion of cases. The cases in which there is a real difficulty in the diagnosis are those in which the disease is complicated with other cutaneous affections, and presents the mixed characters of different eruptions. The cutaneous affections to which it bears more or less resemblance, and from which, in practice, it is to be distinguished, are impetigo, psoriasis, lichen, a form of ptyriasis, scabies, favus. To consider the differential diagnosis with respect to these several affections, would render it necessary to anticipate a description of the distinctive characters entering into the natural history of each. Bearing in mind the characteristic traits of eczema, I shall point out the circumstances which negatively are involved in the discrimination, hereafter, in connection with the consideration, respectively, of the affections just mentioned.

With respect to the particular anatomical element of the skin specially involved in eczema, opinions are not uniform. Rayer regards it as an affection of the follicles. Cazenave, with more reason, thinks that it is seated in the sweat ducts. The red points so characteristic of the disease he supposes to be the inflamed orifices of these ducts. This is probable, but not established beyond doubt.

Like most of the cutaneous eruptions, this undoubtedly proceeds from some ulterior morbid condition; in other words, from a constitutional disorder, which here, as in other instances, scientific research has not as yet unfolded and explained. Whatever may be the internal pathological change, it is usually not accompanied by any marked symptoms irrespective of those which pertain to the local affection. In fact, so far as concerns any appreciable evidences of general or constitutional disorder, persons attacked with eczema, in the majority of cases, seem to be in good health, and to continue in good health notwithstanding the existence of the local affection. Moreover, in much the larger proportion of instances, the subjects of the disease are persons with good constitutions.

From what has just been stated, it follows that our knowledge of the causation of eczema must be far from complete. Some facts, however, are to be stated under this head. It occurs so often in children during dentition, and weaning, that it evidently sustains some relation to these events of infantile life. Under these circumstances its occurrence is by no means sufficient evidence of a strumous taint, although it is perhaps oftener met with in scrofulous subjects. After infancy, young persons are more liable to the disease than the aged. The larger number of cases occur between twenty-five and forty-five.* It not infrequently occurs in females during pregnancy, and is often connected with derangement of the menstrual function. It is not to be regarded as a contagious affection, although occasionally instances appear to favor the opinion that it may possibly be communicated from one person to another. It is apt to affect different members of a family simultaneously, or in succession; but the most rational explanation of this fact is, that they have all been equally exposed to some unknown cause. Cases, especially among children, are much more frequently observed in some seasons than in others. Thus, in the city of Buffalo, in 1845,† it was a matter of common remark among the physicians of that place, that eczematous eruptions were singularly rife. From this fact the possibility of an epidemic predisposition is presumable. As already stated, in some instances it appears to arise from the action of local irritating causes. It is probable, however, that in these cases the local causes simply determine the situation of the eruption, and perhaps also the time of its development, the internal or constitutional morbid condition already existing. So far as season exerts any influence in predisposing to the disease, it would seem, from the statistics given by M. Devergie, that Summer and Winter are more favorable for its production than Autumn or Spring. Of 384 cases, 60 occurred in spring, and 28 in autumn; while 127 occurred in summer, and 169 in winter.

As regards the prognosis, it is not a disease involving danger to life, or serious danger to the general health. The most to be anticipated, as a general remark, is, that it will become chronic, and persist for an indefinite period.

Before speaking of the treatment of eczema, I may take occasion to make a few remarks which, with some exceptions, will be applicable to all cutaneous diseases, and their introduction in this connection will save the necessity of repetition hereafter. I have already said (Lecture I.) that the treatment of these diseases is, in a great measure, empirical. As a general rule, when this is true of any disease, it follows that the remedies recommended by different authors and practitioners are numerous and varied. This is emphatically the case with respect to diseases of the skin; and it is often difficult to decide what are the remedies, the merits of which are based on the most satisfactory proof of success- Published results of experience on this point are seldom presented in the shape of deductions from analysis of the facts

---

* Devergie, op. cit.

† And equally so in 1853.—Ed. Jour.

contained in extensive collections of carefully recorded cases—in other words, the therapeutics of this class of diseases is not based on numerical investigations. In selecting empirical remedies, therefore, we have to be guided, in addition to the notions formed from our own experience, by the testimony of those who have had the largest opportunities for experimental observations, and in whose judgment and integrity we have most confidence. Such is the obstinate character, however, of many of these diseases, that we often have abundant opportunity to make trial of a variety of remedies in the same case.

The principles of treatment in nearly all cases resolve themselves into three divisions, viz:

1st. Obviating suspected remote causes by changing habits, etc.

2d. Meeting the rational indications derived from the local symptoms, such as the degree of inflammation, the existence of excoriation, ulceration, etc., and the general symptoms, or the state of the system irrespective of the local disease. Thus, if there be febrile movement, with fullness of the vessels, and the patient be robust, depletory measures will be indicated whatever be the character of the eruption; but if, on the other hand, the powers of the system are below the standard of health, an opposite course is called for.

3d. Employing entropic or alterative remedies which effect some constitutional change, and also local applications to modify the morbid action of the affected part.

Applying these general principles to the treatment of the affection under consideration, the first point is to search for remote causes. A large proportion of cases occur in infancy, and involve, to a greater or less extent, as we have seen, causes which are appreciable, but not altogether removable. The irritation from teething must continue for some time, but it may be relieved by free scarifications, and division of the gums. The change of diet incident to weaning in some instances involves disorders which we cannot always prevent, but which may be mitigated, if not obviated, by careful adaptation of the food to the power of the digestive organs and the wants of the system. In the treatment of this, and other eruptions in children, exercise in the open air is a measure of great utility. Confinement to heated rooms has appeared to me to contribute to the development of the disease, and I am in the habit of enjoining daily exposure out of doors in all cases occurring in childhood.

Deficient exercise also may enter into the etiology in adults, and without going into details, which would be out of place in this connection, it is sufficient to say that the diet and regimen, both in children and adults, are to be carefully and properly regulated. We are next to endeavor to advance a step farther, from remote, toward proximate causes. The different functions of the body are to be surveyed. Are the digestive organs disordered? Is costiveness present? Is the urinary secretion normal? Does the healthy skin perform its proper functions? These and other similar questions are to guide our investigations, and any functions which may be found to be disturbed, are to be corrected, if practicable, by appropriate means.

We are then to consider the local and general indications. If the patient be of a full habit, the circulation active, the constitution vigorous, and the disease acute, reducing measures, consisting of bloodletting in some cases, and in other cases remedies which deplete by increasing the secretions, viz: cathartics, and diuretics, are indicated, and the diet is to be suited to this condition of the system. But if, on the other hand, the patient be feeble, the body badly nourished, the constitution cachectic or broken down, a plan of treatment directly opposite is to be pursued. Tonics and cordials may be required, and a highly nutritious diet.

The local symptoms, in the acute form of the disease, in its early stage, may indicate local measures to abate inflammatory action, or at all events, the avoidance of applications which will tend to increase this action. But in a later stage local stimulants will often prove important means of cure.

Finally, we may find it necessary to resort to general and local remedies designed to exert in some special manner a curative influence. The general remedies of this class are mercury, sulphur, iodine, alkalies, arsenic, cantharides, etc.

Directing attention somewhat more in detail to the therapeutic measures which may be employed in the management of cases of eczema, we will consider, *first*, the local, and *second*, the general treatment.

The local treatment appropriate to eczema, differs according to the form or species of the disease; to the stage, acute or chronic, and to conditions of the affected surface irrespective of species or stage. In the early stage, and in the acute form, measures, as already stated, to allay the symptoms of inflammation, and exert an antiphlogistic effect are indicated. Warm fomentations and poultices are frequently directed by practitioners under those circumstances, but it is now generally agreed by those who have given special attention to this class of affections, that such applications are injurious save when the disease has become chronic. I have myself seen the local symptoms of simple eczema apparently much aggravated by a flax-seed poultice. If the inflammation be considerable, refrigerant lotions are of service. M. Devergie advises in strong terms irrigations with water, at first slightly tepid, but gradually lowered to the natural temperature, continued from an hour to an hour and a half, once, and sometimes twice daily. This measure is most conveniently resorted to when the extremities are affected. A bucket of water, into which a cock or faucet is inserted, is to be adjusted a little above the part, and the limb placed on India-rubber cloth so disposed as to conduct the water to a vessel, placed below. A piece of cloth is then attached to the faucet and spread over the affected surface; and the water allowed to escape slowly. M. Devergie states that remarkable results sometimes follow these applications, the disease being rapidly deprived of its acuteness. It is, however, only to be resorted to in the summer season, and in persons of good constitutions in whom no unpleasant consequences are to be apprehended from its general refrigerant influence. An emollient application consisting of the *ulmus campestris*, or slippery-elm, ground, and mixed with cold water, I have found useful. Simple cold water applied constantly by means of a linen compress, or lint, produces, by evaporation, a refrigerant effect which in some cases is highly useful. At the St. Louis Hospital whenever cooling lotions are not employed, in the acute stage of this and other cutaneous eruptions, much importance is attached to the application of powdered starch. It is useful by absorbing the irritating fluid which escapes from the vesicles, or exudes from the excoriated surface; but, in addition to this, it is supposed to exert a positive antiphlogistic influence. Ointments or cerates, even of the mildest character, are of doubtful propriety during the acute stage; and all stimulating applications in this stage are injurious.

After the acute stage is passed, other indications are presented. The affected surface is now more or less covered with incrustations, which are speedily renewed after they are thrown off, or their removal effected by means adopted for that purpose. To effect daily the removal of these incrustations is an important part of the local treatment, in order to obviate their irritating effect, and that whatever applications we may wish to make may come into contact with the diseased surface. The crusts are to be removed by prolonged fomentations. A poultice may be applied occasionally for several hours, or, what answers as well, and is more cleanly, the water dressing; and, afterward, the parts bathed, until the morbid deposits are completely removed, with tepid water in which a little alkali (sub. carb. soda or potassa) is dissolved. Soap and water answers the purpose of an alkaline wash. These ablutions are to be repeated twice daily, and care is to be enjoined not to irritate the parts by rubbing, or making forcible attempts to detach the crusts. The alkaline water is to be applied by means of a soft sponge. Considerable patience is requisite on the part of the patient, or friends, in carrying out these directions faithfully, but it is necessary to the successful treatment of the disease. Simple attention to perfect cleansing of the affected part, or parts, in the manner just mentioned, and the use of an absorbent powder, together with attention to diet and a short regimen, will suffice, in many instances, for the cure of eczema in a short period. I have often, especially in children, resorted to these measures alone. Even in cases of long standing, whether in children or adults, in which there has been neglect of cleanliness and hygiene, with perhaps trial of many mercurial applications and internal remedies, it is frequently surprising to observe what may be accomplished in a short time by the mode of treatment already described. This was illustrated before the medical class of the University of Louisville during the winter of 1853–4, in some chronic cases which were treated by me at the Louisville Marine Hospital.

Local applications, however, are undoubtedly necessary to effect a cure in certain cases. Here the question arises, is it always prudent or safe to effect a cure by topical means? Although the fears formerly entertained of the repercussion and translation of these diseases were doubtless exaggerated, we cannot repudiate altogether the correctness of the views on which they were founded; and it seems to me clear that a certain degree of caution is to be observed with reference to this point. Instances of unpleasant sequels of the speedy cure of cutaneous eruptions are too frequent to be attributed merely to coincidence; and the popular notion that the disease, if not manifested on the surface, will be likely to appear elsewhere in some situation less favorable, is not wholly devoid of a pathological basis. Under what circumstances, then, shall local applications designed to arrest the progress of an eruption be made? We cannot answer this question as definitely and clearly as could be desired. We may say that such local applications are admissible whenever the internal morbid conditions which have determined the local affection, have ceased to exist. But often we have no positive knowledge of these conditions beyond the existence of the local affection. Experience tells us that when a chronic cutaneous disease has continued for a long time its very duration enforces caution, as respects the efforts to produce a cure. This is a consideration which renders it desirable not to defer curative applications too long. In general terms we should wait for the results of diet, regimen, and such general treatment as may be rationally indicated, together with the simple local measures that have been already detailed. If this plan prove unsuccessful, local applications with a view to excite a curative process should be employed with a certain amount of circumspection, and frequently conjoined with internal remedies intended to remove the constitutional causes maintaining the eruption.

The local applications designed to be curative act by stimulating, or in some way modifying the morbid condition of the affected surface; those which are found serviceable in chronic eczema, or in other eruptions, are various. I will content myself with merely mentioning some of the most useful, which it is often necessary to employ successively in the same case.

The oxide of zinc, in ointment, is sometimes useful. Mercurial ointments, such as a small proportion of citrine ointment combined with the *unguentum aquæ rosæ*, or cold cream; or calomel contained in simple cerate, will frequently prove efficacious. Whenever an ointment is used, care should be taken that it be not rancid. The ointments prepared by apothecaries are often objectionable on this score. The tar ointment I have in many instances found to produce a very happy effect. At the Hospital St. Louis the empyreumatic oil derived from the juniper berry, called the *Huile de Cade*, is much used in this, and other cutaneous affections. It is applied in some cases pure, and in other instances combined with cerate. Its action is analogous to the tar ointment, but it is supposed to be superior to the latter. So far as I know, this remedy is not as yet in use in this country. It is used as a stimulating and modifying agent in preference to most others, by M. Devergie. Dr. Bennett, of Edinburgh, attaches great importance to the constant application of an alkaline solution, consisting of two drachms of the carbonate of soda, or potash, to a pint of water. A cloth wet with this solution is applied to the affected part, and covered with oiled silk. He assured me that he found this so efficient a mode of local treatment, as to render other applications generally unnecessary.

If the eczema be seated on the scalp the hair should be cut as closely as possible, but not shaved. Without observance of this rule, it is impossible to effect satisfactorily the removal of incrustations, and bring medicinal applications into contact with the diseased surface.

The intense pruritus which is a source of much distress frequently in this affection, claims attention. In their efforts to obtain relief, infants and children sometimes, unless restrained, rub and wound the diseased surface, giving rise to more or less bleeding, and increasing the local inflammation. Camphor, either combined with any ointment that may be used, or in the form of a wash, will in some cases relieve this symptom. A few drops of kreasote added to the ointment, is also useful. The chloroform combined with cerate, is another useful remedy for this purpose. Acetic acid, or vinegar largely diluted is still another.

At, or near the period of cure, the tannin ointment is generally employed at the St. Louis Hospital, with a view to give greater firmness to the newly formed cuticle, and obviate the tendency to relapse. While the skin remains red and glistering, as is often the case for some time, there is constant danger that new vesicles will break out. Light exfoliation of the cuticle is apt to take place for some time after the integrity of the skin is restored. This is true of all cutaneous eruptions. The tannin ointment is supposed to expedite the recovery of the normal tone and vigor of the integument.

The *general*, as well as the *local* treatment varies with the species and stage of the affection. In the acute form at the early stage, sometimes there are present the indications for depletion even by bloodletting. If there be febrile movement, and the patient be an adult, of robust constitution, and full habit, venesection will be proper. In other instances depletion by saline purgatives will suffice. In feeble subjects, and children, of course, neither are judicious. A restricted farinaceous diet is to be enjoined for a short time, except when contra-indicated by existing debility. Acid drinks are useful at this period. M. Cazenave recommends half a drachm of sulphuric or nitric acid in a pint of barley water, to be taken as a beverage.

In the chronic form of the disease numerous remedies are advised. The evidence of their utility is derived from experience, and they are, therefore, using the term in its proper sense, *empirical* remedies. The internal use of alkalies is recommended by several practical writers. Dr. A. S. Morrison advocates particularly the liquor potassæ given in doses of from ten to thirty drops three times daily, administered in the infusion of hops, sarsaparilla or taraxacum. Dr. Bulkley, of New York, speaks highly of this remedy.* The bicarbonate of soda is also used, in doses of from fifteen to thirty grains. The iodide of potash I have known useful, particularly in the case of a patient who suffered from repeated attacks, and in which it was several times followed by a speedy cure when other measures proved inefficacious. The iodide of sulphur is much esteemed by Devergie. Mercury, especially the bichloride, is extolled by some. It is generally, however, given dissolved in the compound decoction of sarsaparilla, or some analogous vehicle in which it undergoes partial decomposition. Arsenic has been found useful in this, but less so than in other forms of cutaneous disease, more especially the squamous affections. What is called Donnivan's solution, which combines mercury, iodine and arsenic, has been much employed in England and this country for several years past, in many obstinate cutaneous eruptions, and in eczema among the number. The tincture of cantharides is another remedy which may be tried, if others fail.

I do not profess to have mentioned all the remedies, local or general, which

---

* New York Journal of Medicine, January, 1846.

have been found useful in the treatment of eczema; but only the more prominent of them. I have not discussed their relative merits, or taken pains to present the evidence on which their claims respectively rest. To undertake to do this would involve a more extended consideration of the subject than the limits of a few lectures will permit. Moreover, in treating of therapeutics here, as in connection with other classes of diseases, to consider at length individual remedies, would be to encroach on the province of my colleague and friend, the professor of materia medica. I shall only aim to present leading general principles, simply enumerating the more important of the particular measures which are recommended by practical writers, and of the value of which I can speak, measurably, from personal experience.

Eczema is, of all cutaneous diseases, the one most frequently met with in practice. This is shown by the following statistics: Of 1800 cases received at the St. Louis Hospital, precisely one-third were cases of eczema.* Of 807 cases treated at the dispensary infirmary of the city of New York during two years, 226 were cases of eczema.†

* Devergie.

† New York Journal of Medicine, July, 1846.

